# Supplementary figures and images for: An image-computable model of human visual shape similarity
Source: PLoS Comput Biol. 2021 Jun 1;17(6):e1008981. doi: 10.1371/journal.pcbi.1008981 (PMC8195351; doi:10.1371/journal.pcbi.1008981)

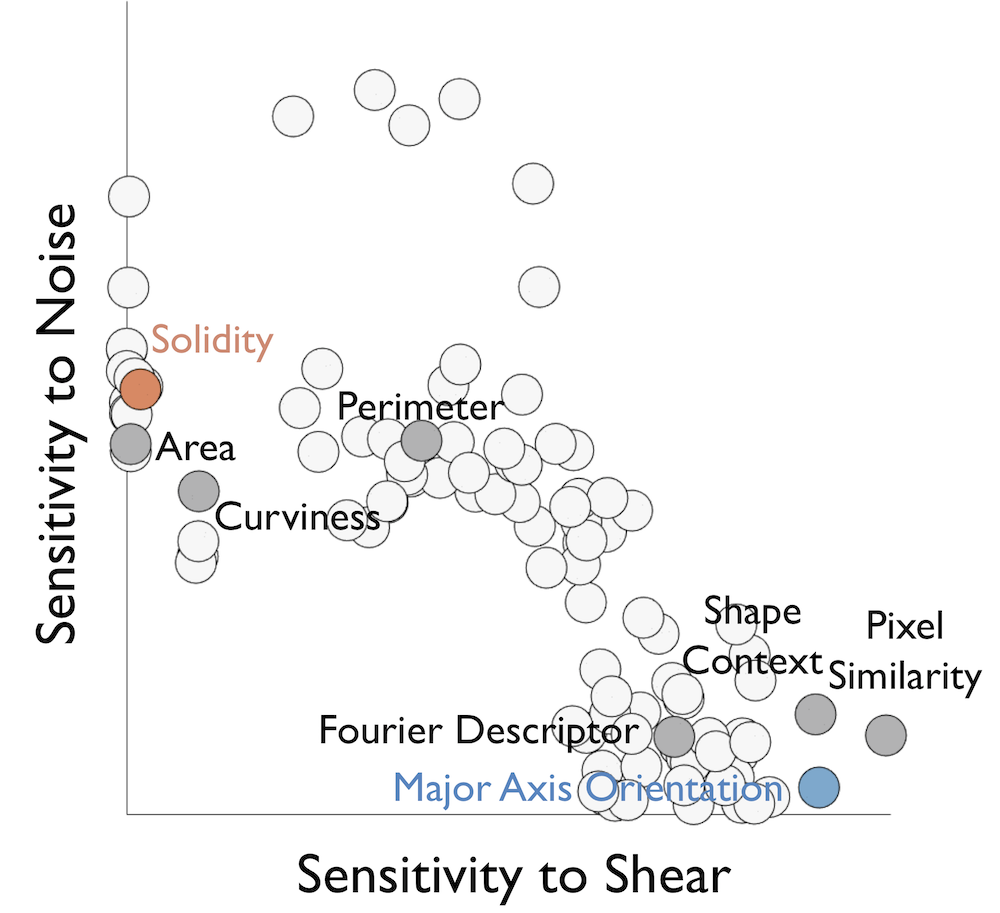

Supplement: S1 Fig — Here, solidity, area, and curviness are more sensitive to noise than shear, while major axis orientation is less sensitive to noise than shear. That different descriptors are tuned to different transformations highlights their complementary nature. (TIFF) [file pcbi.1008981.s001.tiff]

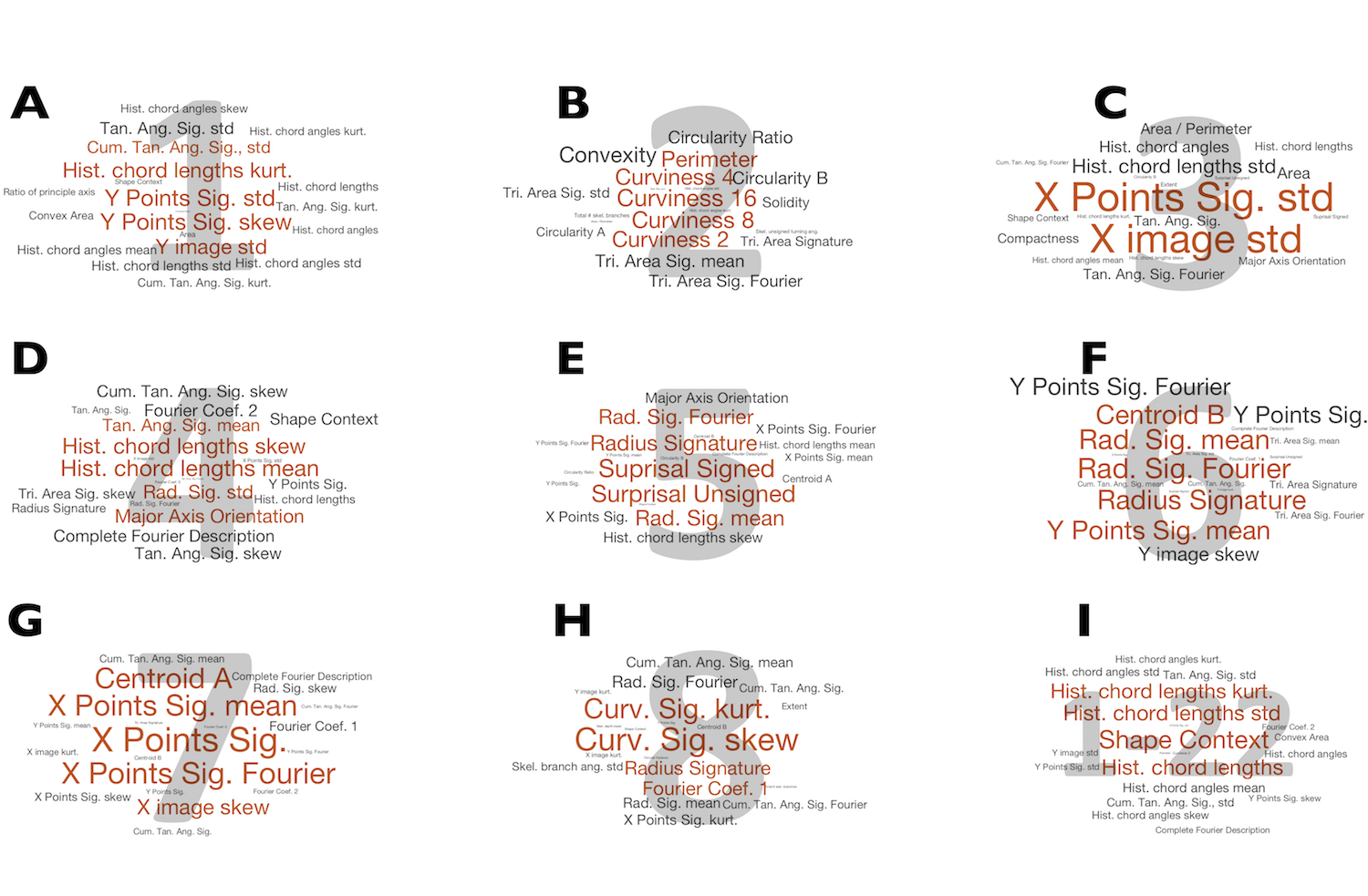

Supplement: S2 Fig — A wordcloud that shows the 20 best features in terms of absolute correlation to each of ShapeComp’s first 8 dimensions (A-H) and (I) across all 22-dimensions. The largest words in the cloud, the most predictive features, are highlighted with colour. (TIFF) [file pcbi.1008981.s002.tiff]

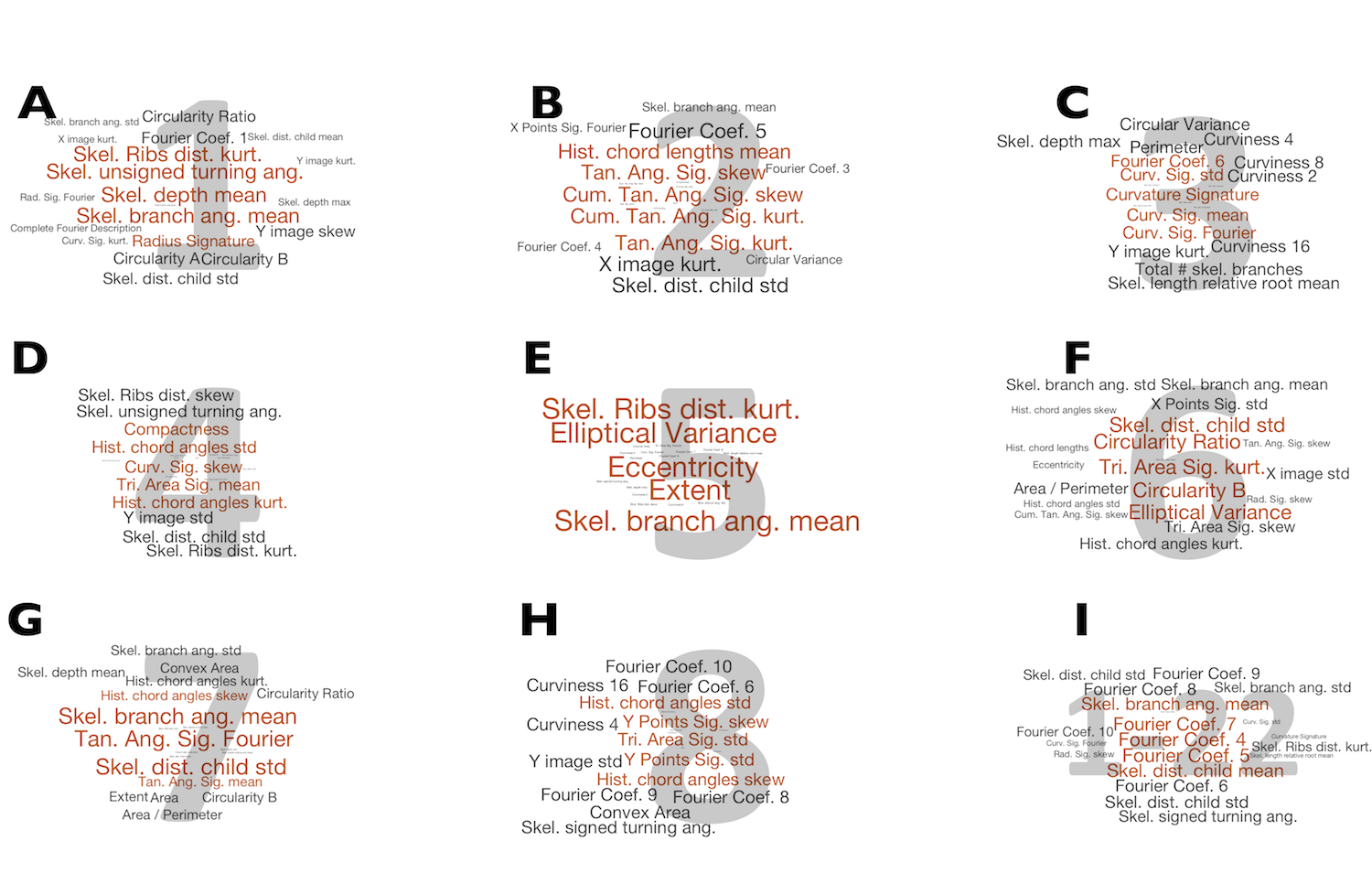

Supplement: S3 Fig — A wordcloud that shows the 20 features that least predictive (in terms of absolute correlation) to each of ShapeComp’s first 8 dimensions (A-H) and (I) across all 22-dimensions. The largest words in the cloud, the least predictive features, are highlighted with colour. (TIFF) [file pcbi.1008981.s003.tiff]
