# Supplementary material for: An image-computable model of human visual shape similarity
Source: PLoS Comput Biol. 2021 Jun 1;17(6):e1008981. doi: 10.1371/journal.pcbi.1008981 (PMC8195351; doi:10.1371/journal.pcbi.1008981)
Supplement: S1 Table — (DOCX) [file pcbi.1008981.s004.docx]

**Supplemental Tables**

**Table S1. List of 109 shape descriptors in ShapeComp.**

| **Descriptors** | **Additional Comments** |
| --- | --- |
| **Simple shape descriptors** | Motivated by Zhang and Lu [1], Paulun et al. [2], and Peura and Iivarinen [3]. Many of these descriptors were computed with MATLAB’s regionprops command. Computations for (1)-(22) are based on image representation of shape. (23-109) are based on point representation of shape. (27) and (28) are computed with help of geom2d toolbox (by David Legland) from the MATLAB file exchange. |
| (1) Area | regionprops |
| (2) Perimeter | regionprops |
| (3) Eccentricity | regionprops |
| (4) Major Axis Orientation | regionprops |
| (5) Ratio of principle axis | Major/minor axis length |
| (6) Extent | regionprops |
| (7) Compactness | ^^ |
| (8) Solidity |  |
| (9) Circularity A | 4π(Area/Perimeter^2^); from Paulun et al. [2] based on regionprops output. |
| (10) Circularity B | Perimeter^2^ /Area ; from Zhang and Lu [1] |
| (11) Convexity | Ratio of perimeters of the convex hull over that of the original contour |
| (12) Centroid A | X-coordinate of shape centroid in image (from Paulun et al. [2]) |
| (13) Centroid B | Y-coordinate of shape centroid in image (from Paulun et al. [2]) |
| (14) Circularity Ratio | Area of shape to area of circle, where the circle has the same perimeter |
| (15) Convex Area | Paulun et al. [2] regionprops |
| (16) Area/Perimeter | Zhang and Lu [1] |
| (17-19) Statistics for horizontal (x) distribution of image intensities | Standard deviation, skewness, kurtosis; from Paulun et al. [2] |
| (20-22) Statistics for vertical (y) distribution of image intensities | Standard deviation, skewness, kurtosis; from Paulun et al. [2] |
| (23-26) Curviness at different scales | RMS error of shape to blurred version of itself. Blurred versions were filtered version of original shape with kernel size of 2, 4, 8, or 16 points. Based on Paulun et al. [2] |
| (27) Circular Variance | Peura and Iivarinen [3]  The proportional mean square error with respect to a circle of the same area |
| (28) Elliptical Variance | Peura and Iivarinen [3]  The proportional mean square error with respect to an ellipse of the same area |
| **Shape Context** | Belongie and Malik [4] |
| (29) Shape Context | A joint histogram of distance (bins = 15) and angle (bins = 15) |
| (30)Histogram of chord lengths | bins = 100 |
| (31) Histogram of chord angles | bins = 100 |
| **Shape Signatures** | Motivated by Zhang and Lu [1]. |
| (32) Radius Signature |  |
| (33)Curvature Signature |  |
| (34) Triangular Area Signature |  |
| (35) Tangent Angle Signature |  |
| (36) Cumulative Tangent Angle Signature |  |
| (37) Horizontal (x) distribution of points |  |
| (38) Vertical (y) distribution of points |  |
| **Frequency Decomposition** |  |
| (39 - 48) Coefficients 1 to 10 | Separate descriptors for first 10 coefficients; Kuhl and Giardini [5] |
| (49) Complete Fourier Description | Kuhl and Giardini [5] |
| **Surprisal** | Information, in Shannon’s sense, along contours  Feldman and Singh [6]. |
| (50) Signed |  |
| (51) Unsigned |  |
| **Boundary Moments** | Mean, standard deviation, skew, and kurtosis of (30-38). Motivated by Zhang and Lu [1]. |
| (52-55) Statistics for histogram of chord lengths |  |
| (56-59) Statistics for histogram of chord angles |  |
| (60-63) Statistics for radius signature |  |
| (64-67) Statistics for curvature signature |  |
| (68-71) Statistics for triangular area signature |  |
| (72-75) Statistics for tangent angle |  |
| (76-79) Statistics for cumulative tangent angle |  |
| (80-83) Statistics for x-values |  |
| (84-87) Statistics for y-values |  |
| **Shape Skeleton** | Feldman and Singh [7]  Wilder, Feldman, and Singh [8]  Computed with ShapeToolbox |
| (88) Total number of skeletal branches |  |
| (89) Maximum depth of skeleton |  |
| (90) Mean skeletal depth |  |
| (91) mean branch angle |  |
| (92) mean distance along each parent axis at which each child stems |  |
| (93) mean length of each axis relative to root |  |
| (94) total absolute unsigned turning angle integrated along the curve |  |
| (95) total (absolute value of) signed turning angle of each axis integrated  along the curve |  |
| (96) standard deviation of (91) | To our knowledge, skeletal summaries (96-102) have been previously used to describe shape. |
| (97) standard deviation of (92) |  |
| (98) standard deviation of (93) |  |
| (99 – 102) Statistics for distribution of ribs | Mean, standard deviation, skewness, kurtosis |
| **Fourier Component** | Fourier descriptions of shape signatures from (32-38) in terms of absolute value of one-sided Fourier component |
| (103) Fourier transform of radius signature |  |
| (104) Fourier transform of curvature signature |  |
| (105) Fourier transform of triangular area signature |  |
| (106) Fourier transform of tangent angle signature |  |
| (107) Fourier transform of cumulative tangent angle |  |
| (108) Fourier transform of distribution of X values |  |
| (109) Fourier transform of distribution of Y values |  |

**Supplemental References**

[1] Zhang, D., & Lu G. (2004). Review of shape representation and description techniques. Pattern Recognition, 37, 1-19.

[2] V.C. Paulun, T. Kawabe, S.Y. Nishida, R.W. Fleming, Seeing liquids from static snapshots. Vision research, 115, 163-174, (2015).

[3] Peura, M. & Iivarinen, J. (1997). Efficiency of simple shape descriptors, in: Proceedings of the Third International Workshop on Visual Form, Capri, Italy, May, pp. 443–451.

[4] Belongie, S. & Malik, J. (2000). ["Matching with Shape Contexts"](http://ieeexplore.ieee.org/iel5/6885/18538/00853834.pdf). IEEE Workshop on Contentbased Access of Image and Video Libraries (CBAIVL-2000).

[5] Kuhl, F. P., & Giardina, D. R. (1982). Elliptic Fourier features of a closed contour. Computer Graphics and Image Processing 18: 236–258.

[6] Feldman and Singh (2005). Information along contours and object boundaries. Psychological Review. Vol 122. No. 1, 243 -252

[7] Feldman, J., & Singh, M. (2006). Bayesian estimation of the shape skeleton. Proceedings of the National Academy of Sciences, 103(47), 18014-18019.

[8] Wilder, J., Feldman, J., & Singh, M. (2011). Superordinate shape classification using natural shape statistics. Cognition, 119, 325–340.
