# Supplementary material for: An image-computable model of human visual shape similarity
Source: PLoS Comput Biol. 2021 Jun 1;17(6):e1008981. doi: 10.1371/journal.pcbi.1008981 (PMC8195351; doi:10.1371/journal.pcbi.1008981)
Supplement: Attachment — Submitted filename: ShapeComp-R1-01-ResponseLetter.pdf [file pcbi.1008981.s005.pdf]

REVISIONS TO MORGENSTERN et al. PLOS COMPUTATIONAL BIOLOGY

We thank the reviewers and editors for their helpful comments.

Here, to indicate changes we have made to the MS, new next is highlighted in red. Reviewers' comments are italicized, and our responses are indented and in normal type.

Editor's comments

Thank you very much for submitting your manuscript "An image-computable model of human visual shape similarity" for consideration at PLOS Computational Biology. Your manuscript was reviewed by members of the editorial board and by three independent reviewers. As you will notice in the reviews (below this email), the Reviewers are generally positive about the model, but they have mixed opinions about the ultimate contribution of this study. While Reviewers #1 and #2 believe that the study "will be of interest to many scholars in the field" and "represents an important advancement in the field", respectively, Reviewer #3 states that "the conclusions drawn in the manuscript [are] restatements of things that we already know".

In light of the reviews, we would like to invite the resubmission of a significantly-revised version that takes into account the reviewers' comments. It will be of particular importance to spell out more clearly the study's contribution to the field, viewed from the perspective of the readership of PLOS Computational Biology.
We cannot make any decision about publication until we have seen the revised manuscript and your response to the reviewers' comments. Your revised manuscript is also likely to be sent to reviewers for further evaluation.

> Dear Editors, thank you for allowing us the opportunity to address the reviewers concerns. We have added several experiments, and significantly edited the MS to clearly state the main contributions of the paper by highlighting the empirical contributions in the introduction. To date, there is no data-driven image-based model predictive of human shape similarity. We believe our paper highlights the usefulness of data-driven approaches and models in accounting for perceptual phenomena, and will be useful as a standard to many scientists in numerous fields. We also note the PLOS computational biology has a history of publishing similar standards and tools (e.g., A toolbox for representational similarity analysis, Nili et al, 2014). Please see below the responses to reviewers.

Reviewer 1's comments

The authors define a model that combines information from many different shape representations and show how it can be used to explain human shape similarity judgments. Their model is very comprehensive and represents an important advancement

in the field. Other recent attempts to define a shape space have been to simple and too constrained to small sets of images and specific tasks to be relevant for understanding the full complexity of the shapes of our world and the wide variety of tasks that involve interacting with them.

Overall, the authors do a good job of describing the use of the model, and referring the reader to the components of the model.

The only part I find lacking is any discussion about dimensionality reduction. The authors use MDS to reduce the number of dimensions from 109 to 22. These 22 dimensions are combinations of the original 109. I would have liked to know more about the usefulness of many of the original 109 dimensions. Are there any that are not worth including? Can any be removed without affecting the 22 dimensional-space that gets recovered? Which of the original 109 dimensions load most strongly onto the 22 dimension? Can anything be said about which of the original dimensions are most important to include?

Thank you for the suggestion. By comparing the original 109 features to ShapeComp's 22 dimensions, we have now added new supplementary figures and a discussion of which features are most important for ShapeComp (on page 11):

Given these 22-dimensions are composites of the original 109 features, one might ask what are that best original features? **Figure S2 A-H** shows that several of the original features are highly correlated to each of first 8 dimensions of ShapeComp (which already accounts for greater than 85% of the variance in animal shapes), suggesting that many features tap into complementary aspects of shape. Thus, ShapeComp will not undergo major changes if one of the original features is removed. Similarly, **Figure S3 A-H** shows several poor predictors that presumably change less across the animal silhouette database than changes in other features. **Figures S2I** and **S3I** show the best and worst features across the full 22D space, respectively. The Shape Context and summaries based on the Shape Context (e.g., histogram of chord lengths) were most predictive of ShapeComp, while the skeletal and low frequency Fourier descriptors were least predictive. (Note, however, the less predictive shape descriptors are likely still useful for shape similarity. Firstly, the features posited here are partial summaries of the original shape descriptors. For example, one feature taken from the shape skeleton was the number of ribs. There are likely a number of other ways to summarize the shape skeleton that may be more sensitive to change in animal shapes across our database. Secondly, it is likely that such features play an important role in finer shape discrimination judgments that go beyond ShapeComp's 22-dimensions.)

We also note that since several of the original features are highly correlated to each ShapeComp dimension, ShapeComp is somewhat robust when it is recomputed based on a subset of 55 of the 109

dimensions (Figure 2F) and on different random subsets of animal shapes (Figure 2F) suggesting that removing features will not have a major effect on ShapeComp's underlying space.

Furthermore, we find that ShapeComp is fairly robust against removal of the features that are best related to ShapeComp (i.e., the 11 features related to Shape Context and its summaries), showing high correlations between the full space and the space with fewer features (*r* = 0.77, *p*<0.01). We add this result on page 12:

In addition, despite removing the most predictive features of ShapeComp (i.e., 11 features related to the Shape Context and its summaries; listed as descriptors 29-31, and 52-59 in **Table S1**), pairwise distances between shapes remain highly correlated (*r* = 0.77, *p*<0.01). Thus, ShapeComp appears to capture a high-dimensional understanding of shape that tends to be somewhat independent across the specific selection of animal shapes or even shape descriptors.

Following this line of thinking I was wondering if the authors tried to use the CNN to recover the original 109 dimensions, or only the 22 dimensions. Beyond having the reduced dimensions, is there something about the 22 dimensional space that makes it easier to accurately determine the ShapeComp representation? Or would it be just as easy to compute the 109 dimensions and use the same transformation to reduce it to the 22 dimensions?

For several reasons, we only used the CNN to recover 22 dimensions from MDS rather than the original 109 dimensions. First, the 22 dimensions from MDS (in ShapeComp) reduce redundancy that is coded from the original descriptors. A change in shape area, for example, is highly correlated to a change in its perimeter. Thus, we probably don't need both of these descriptors, and MDS eliminates the redundancy between them leading to a more efficient and desirable representation where the 22 dimensions now vary independently from one another. Of course other dimensionality reduction techniques could also be used, and these would also be an improvement over the raw, original 109-dimensional space. A second reason that we did not train a CNN on the original features is that there are a priori reasons for doubting that the visual system actually computes these specific features. Instead, we used these features as a means to capture distinct (complementary) sources of variation across shapes. Furthermore, the process of dimensionality reduction can also be thought of as explicitly testing the extent to which different metrics can capture variations in natural shape. Features that measure irrelevant properties end up contributing only small weights to the retained dimensions, so we are excluding them from further consideration. The analysis presented in response to the previous comment addresses this perspective.

As computing the 109 dimensions using a collection of shape processing tools would be extremely burdensome, the inclusion of the CNNs that can give similar enough results with a single tool is very helpful. The authors do not explain how to make use of each component, so it would be difficult to reproduce this work, and would require looking back at many different articles, however, while I would normally find this problematic, but releasing a CNN that can do something similar allows for the community to benefit from the authors' work.

Overall, I find these results important and useful for the field. This work was an immense undertaking and the field will benefit from its publication.

Reviewer 2's comments

The manuscript describes a model which uses 109 shape descriptors from the scientific literature to predict human shape similarity judgments between pairs of shapes. The model has a number of significant strengths (documented throughout the manuscript) and, of course, some weaknesses (kudos to the authors for nicely describing these weaknesses toward the end of the manuscript).

I have mixed feelings about this manuscript but, ultimately, I believe its negative features outweigh its positive features.

In brief, the manuscript attempts to advance our understanding of human visual shape perception. However, it never tells the reader the new and important insights provided by this research. Indeed, I found the conclusions drawn in the manuscript to be restatements of things that we already know. Consequently, I don't feel as if I learned anything new on the basis of this research.

For example, a major conclusion of the manuscript is stated as follows (page 7): "More generally, the plot shows the wide range of sensitivities across different shape metrics, indicating that depending on the context or goal, different shape features may be more or less appropriate." I agree completely. But I (and nearly all other researchers in the field) knew this already. Does the reported research shed any new light?

> We did not mean for this to be interpreted as a "major conclusion" in our paper. It's part of the introduction to motivate the approach for a general readership. The point is that while individual features have complementary strengths, combining them allows more powerful representations. We are unaware of any previous study that explicitly quantifies the relative robustness and sensitivity of so many different shape similarity metrics as we have done. But again, it is just a didactic/illustrative point in the intro.

As a second example, the manuscript concludes (page 10): "Thus, while not all 109 shape descriptors are independent, a multidimensional space is indeed required to capture the variability inherent in animal shapes." Again, I agree completely. But, again, everyone in the field already knew this. I've never yet met a researcher in the field that thought that

visual shape is simple, so simple that a one-dimensional space suffices to capture the variability inherent in animal shapes. Again, it seems important to ask if the research reported here sheds any new light?

> See a fuller response below. We have removed the offending sentence. It was not intended to be a major point.

As a third example, the manuscript concludes (page 19): "Consistent with previous works [44, 79-85, 87], this confirms that human shape similarity relies on more sophisticated features than pixel similarity alone." Yes, of course. Everyone in the field already believes this. Was there some doubt in the field, thus requiring further investigation?

I could go on and on. Here are a couple of more quotes from the manuscript. On page 20, the manuscript states, "This indicates that human shape perception relies on more than a single ShapeComp dimension." On page 20, the manuscript states, "Together, these results show that human shape similarity relies on multiple ShapeComp dimensions-- highlighting the importance of combining many complementary shape descriptors into ShapeComp." Yes, of course. I agree with all these statements and many more too. So does everyone else in the field. I wish the manuscript told us the new and important insights provided by the authors' research.

(As an aside, if it seems as if I'm repeating myself here, it is because the manuscript is very repetitive. I estimate that it could be cut by 40%-50% without loss of meaningful content.)

> We certainly agree with the reviewer that most researchers think shape representations involve more than one dimension, and that pixel similarity is not the best way to capture perceptual similarities in shape.
>
> And yet, although everybody supposedly agrees this, it is striking just how often such crude 1D and/or pixel-based metrics are used in the literature to compute shape similarity. In fact, this is the norm, not the exception (we cite *numerous* examples in the paper). Many of the individual features we include in the ShapeComp model were originally proposed as shape similarity metrics *on their own*. So, while people allegedly believe that shape is multidimensional, this often (usually?) doesn't translate into concrete models of shape similarity actually employed in the literature.
>
> The continued reliance on such crude metrics reflects a lack of any standard alternative model. So, there is clearly a significant unmet need among researchers in human and machine vision for an image-computable, multidimensional shape similarity metric. Providing such a model is the primary contribution of this work. This is indicated in the title of the MS, which does not refer to novel findings about how shape is represented in the human visual system, but to the model itself.

We have created and shared a concrete implementation that has the desired characteristics, which everyone can now use. It's true that we then also verified that the model does indeed have the desired characteristics (e.g., surpassing simplistic pixel-based metrics), and confirm that human shape perception is indeed multidimensional. But to focus on this is to miss the main contribution: there is now a data-driven, image-computable model that people can apply to their research, rather than just alluding in vague verbal terms to some unspecified and unknown number of dimensions.

Moreover, most previous shape similarity metrics have been posited based on intuitions, rather than in a data-driven way—i.e., derived from or related to the statistics of natural shapes. We believe that with a data-driven approach, ShapeComp provides a useful baseline for many different applications, and across disciplines.

We are convinced that this is a valuable contribution in its own right. Of course, future work should use this tool to gain deeper, novel insights into human visual shape processing and its neural underpinnings. But we feel the model and its validation is already complex enough to warrant publication as it stands.

Having said all that, we take on board the reviewer's comment. We clearly did not do a good enough job of setting up the motivation of our work and its intended contribution. To address this, we have made numerous changes throughout the MS. We have also tried to find places to cut, although, 40% was not achievable without heavily compromising either readability or content.

A few of the changes include:

(1) Altering the abstract to reduce the impression that are claiming the idea of multidimensional representations as a novel contribution

(2) Adding the following text to the introduction:

The key idea motivating our model is that human vision may resolve the conflicting demands of robustness and sensitivity by representing shape in a multidimensional space defined by many shape descriptors (**Figure 1B)**. While it is widely appreciated that visual shape representations are likely multidimensional, in practice computational implementations of shape similarity metrics have typically used only a small number of quantities to capture relationships between shapes (Ons, De Baene, & Wagemans, 2011; Huang, 2020; Wilder, Feldman, & Singh, 2011). As opposed to previous work, here we provide a data-driven implementation that produces the dimensions needed to distinguish natural animal shapes. The approach does in fact contain many more dimensions than proposed previously, sufficiently accounts for human shape similarity,

and provides a novel baseline metric against which more sophisticated computations can be compared.

(3) Changing wording in many places to clarify that our results are *confirming* that the model fulfills expected characteristics (i.e., the findings are not intended to be novel claims about the functioning of the visual system, but verification that the model behaves as expected)

(4) Adding the following text to the General Discussion:

While it is widely believed that human shape representations are multidimensional, to date there has been no comprehensive attempt to implement this idea in a concrete image-computable model. Moreover, the continued widespread use of relatively simplistic pixel-based similarity measures [52, 73-87] points to a significant unmet need for a standard alternative model. The main contribution of this study is to provide such a model.

Here are a few other comments that may be helpful to the authors:

The authors should keep in mind that shape similarity is generally thought of as a means toward an end, not as an end in itself. For instance, shape similarity estimates might be useful in a system that performs visual object recognition, a system that plans motor movements for grasping, a system that performs problem solving and action planning, etc. I encourage the authors to use their model for one of these applications, and then write a manuscript about the great performance of their system (relative to other systems).

We fully agree with the reviewer about the role of shape similarity in multiple tasks, and indeed use exactly this observation to motivate our study in the opening sentences of the MS.

Indeed, our broader research program investigates the role of shape representations in many such domains, as documented in numerous publications in recent years (see, e.g., our recent publications on the role of shape in material perception in Current Biology and PNAS; in grasping in PLoS Computational Biology and in object categorization in Cognition, Cognitive Psychology, Scientific Reports and other venues).

As the reviewer rightly points out, it goes without saying that we will use the ShapeComp model in future studies to investigate shape perception in such domains as one-shot learning, mapping shape representations in the ventral stream, predicting grasp parameters, inferring causal history, perceiving material properties, identifying animacy in novel objects, determining functional properties of object (affordances), predicting

future object behaviour, etc, etc. We also hope others use ShapeComp to investigate questions that we haven't thought of yet.

However, we disagree that such studies would only be worth publishing if ShapeComp has 'great performance' relative to other systems. The model is meant to be a baseline metric, against which more sophisticated computations that represent deeper underlying generative processes can be compared. It is a simple, feedforward model. It's multidimensional and data-driven, unlike most previous work, but still a purely discriminative approach to capturing shape similarity. Yet we believe that providing such a baseline model is valuable in its own right.

To appreciate this, perhaps the following analogy is useful: in imaging studies, phase scrambling is often used to control for low-level stimulus characteristics. Nobody thinks that images are represented only as amplitude spectra, yet, phase scrambling is certainly a better way of controlling for low-level image characteristics than, say, pixel scrambling.

In a similar way, ShapeComp provides a better baseline measure of shape similarity than the widely used IoU metric. It is a tool for objectively measuring how visually similar two shapes are without having to run an experiment. This is really useful in its own right! It will accelerate progress on research in the same way as visual difference predictors have accelerated progress on image quality metrics and a host of other areas.

The manuscript mentions one or two applications of the model toward its end. For instance, the manuscript shows how the model can be used to derive perceptually uniform shape spaces of novel objects. I agree that this might be useful for experimentalists in the vision sciences. However, without careful comparisons, there is no way of knowing whether the method described here is better or worse than its alternatives.

To our knowledge, ours is the first article to propose an automatic means to generate perceptually uniform shape spaces of complex novel objects. It seems to us that the onus is on the developers of alternative methods to demonstrate that they are better or worse than ShapeComp at achieving this goal.

Nevertheless, we have now added an analysis that compares ShapeComp to the current standard model of object processing which predicts human behaviour, brain imaging, and neurophysiology. In Figure 11 we show that ShapeComp is much more predictive of human shape similarity than these object recognition CNNs suggesting that at least for studying shape coding in the brain, or creating perceptual similar shape spaces, ShapeComp would be a better model.

Lastly, the manuscript states that a limitation of the proposed model is that it only considers 2D shape descriptors, whereas "for many applications it would be desirable to

characterize similarity in 3D" (page 34). I fully agree. To me, this seems like a great area that is ripe for new and important insights.

Thank you. We plan to go in this direction in the future.

Reviewer 3's comments

In this manuscript, the authors developed (and tested) a model to capture the human ability to perceive shape similarities. Overall, the results are very promising and suggest that human ability to perceive shape similarities can be explained by combining a large number of shape dimensions and that multiple dimensions better perform over and above every single dimension or shape silhouette. The model ('ShapeComp') reaches human-level performance with real-world object shapes (animal shapes) but also when using novel shapes, and across a wide range of tasks (e.g., similarity judgments across pairs of shapes, multiple object similarities). The results appear very promising despite some limitations, partially already discussed in the manuscript.

The study tackles a relevant problem across several research fields from computer vision to cognitive neuroscience and goes one step further by combining a large number of shape descriptors in trying to capture the human (special) ability to perceive the shape. Many are the models that have tried to address this question mainly focusing on one or two dimensions. Together, these results further support the multidimensional nature of human perception and highlight the limitations of focusing on one/few single dimensions to describe shape perception. This study will be of interest to many scholars in several fields.

I enjoyed reading this manuscript and I appreciated particularly the critical approach taken by the authors. While reading this work, I posed myself several questions, which happen to be addressed one by one in the subsequent sections of the manuscript. For instance, based on Figure 2A, I asked myself whether much simpler models such as a simple shape silhouette would be enough to capture most variance in the data; I was happy to see that the authors also considered this control analysis. I also appreciated the subsequent controls to rule out the contribution of individual descriptors. In addition to this, I feel it would be relevant to test the ShapeComp against the performance of recent deep neural network. For instance, it has shown that pre-trained DNNs are good models to approximate human ability to perceive shape (Kubilius et al., 2016). It would be particularly relevant to test to what extent the ShapeComp model can better explain human shape perception relative to standard pre-trained DNNs.

Thank you for your comments and interest in the paper. Comparing ShapeComp to standard DNNs is an interesting test, as some work shows that DNNs are good models for approximating human shape perception (Kubilius et al., 2016), while other work argues that these networks rely on texture much more than shape (Gerhois et al., 2019). We compared ShapeComp's ability to standard DNNs and add it into our results section (on pages 32-34):

ShapeComp predicts human shape similarity better than object recognition convolutional neural networks (CNNs) for novel shapes

Although shape is thought to be the most important cue to human object recognition, its role in artificial CNN object recognition is less clear. Some work observes that the networks are good models of human shape perception (Kubilius et al., 2016) while other studies note that conventional CNNs have some access to local shape information in the form of local edge relations, but they have no access to global object shapes (Baker, Lu, Erlikhman, and Kellman, 2018), and are typically biased towards textures (Geirhos et al., 2019). Kubilius et al. (2016), showed that GoogLeNet (Szegedy et al., 2015) is highly consistent with human object categorization based on shape silhouette alone, and showed how similarity in the outputs from its last layer clearly groups such silhouettes into object categories (e.g., man-made versus natural). It is therefore interesting to ask how well such object recognition neural networks predict human similarity judgments of novel objects like those we used for testing our participants and the ShapeComp model.

We tested this by deriving predicted shape similarity from various pre-trained networks, for the novel GAN shapes from our rating experiment (in **Figure 5b**, from *Distances in ShapeComp model predict human shape similarities for novel objects*) and our similarity arrangements (in **Figure 7**, from *Deriving perceptually uniform shape spaces of novel objects*). Following Kubilius et al. (2016), we defined network shape similarity as Euclidean distance in their final fully-connected layer (with 1000 units). We find all the networks we considered were substantially less predictive of human shape similarity than ShapeComp, both in pairs of shapes and across sets of shapes (Figure 11). ShapeComp, was much better at predicting human shape similarity than GoogLeNet in pairs of novel shapes (Figure 11A) and across shape sets (Figure 11B), highlighting fundamental differences in the computation of shape by object recognition neural networks and humans. Even the best performing of the networks we tested (Resnet101) correlated poorly with human judgments compared to ShapeComp, despite its vastly larger feature space. Together these findings suggest that the ability to label objects in natural images is not sufficient to account fully for human shape similarity judgments. We speculate that the nature of the shape computations in supervised object recognition neural networks trained on thousands of natural images is likely one of the many reasons why they fail to generalize like humans do, often incorrectly classifying cartoon depictions of images that even children with little experience easily classify. Consistent with this idea, increasing shape bias in these object recognition networks improves their accuracy and robustness (Geirhos et al., 2019).

The authors used real-word shapes of animals (> 25000) as input information. I appreciate this choice. As the authors also mention, this specific choice was driven by the consideration that human vision is exposed (since birth) to real-world shapes of meaningful objects. Therefore, a model of human shape needs to account for those perceptual biases driven by high-level aspects such as object meaning, class, or context that humans will probably try to extrapolate even when confronted with novel shapes. For this reason, I think it would be useful to test whether this model can capture intrinsic biases that might shape human shape perception. For instance, if I understand correctly from the methods section, in the analysis reported in figure 2B and 2C the authors tested the ability of ShapeComp to capture human similarity judgments in two sets of shapes within the same animal class (houses and rabbits). It would be interesting to test whether this result generalises to subsets of images that span a few animal classes. Would humans use the same strategy in both situations: when asked to arrange shapes for objects (e.g., animals) within the same class vs multiple classes? Or would the latter setting result in a shape arrangement that does also take into account high-level object information? Since human perception does not happen out of context, this test would be relevant when trying to approximate human shape perception.

We added a new experiment that compares human similarity (n=10) and the full model across 5 categories of animal shapes with 6 shapes each (30 shapes total). The results are now reported in Figure 2C and E. ShapeComp still predicts the global relation between shapes. For example, looking at Figures 2C and E, in both humans and ShapeComp, spiders are closer to horses than rabbits, turtles are nearest to rabbits, and elephants are in between rabbits and horses.

Semantics, however, does play a role; ShapeComp still preserves the relationship between the human arrangements of shapes (*r*=0.63, which is a small decrease from *r* = 0.69 that we previously reported within categories). Despite this minor decrease, ShapeComp still predicts shape similarity of familiar shapes better than object recognition networks previously shown to characterize shape similarity in familiar shapes (Kubilius et al., 2006). ResNet101, for example, which we find best accounts for shape similarity, only weakly accounts for human arrangements of animal shapes across the 3 sets (*r* = 0.2, see figure below). (Note that this correlation cannot be directly compared to Kubilius et al. who looked at consistency with humans in terms of categorization from the object's silhouette.)

This experiment also reinforces our initial reason for using GAN shapes, which is that similarity of familiar shapes is heavily influenced by semantics. Thus, to create a proper benchmark for geometric features rather than semantic attributes, novel shapes like GANs provide a better stimulus set.

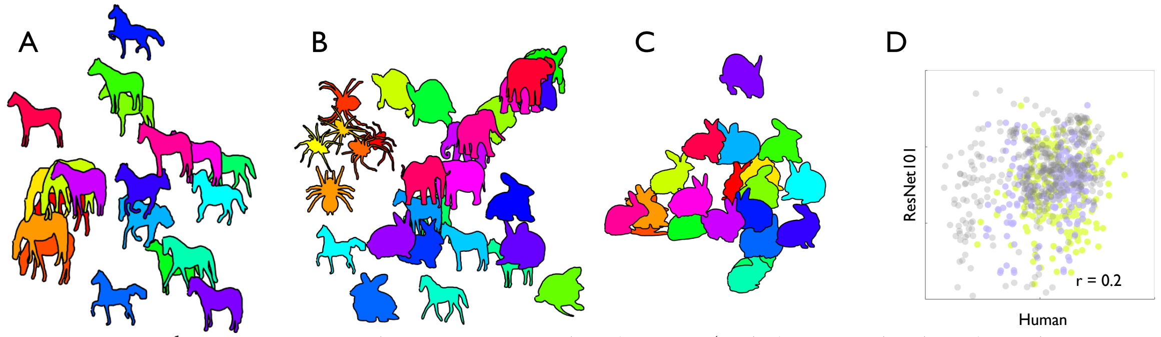

Figure shows ResNet101 similarity arrangements based on MDS (multidimensional scaling; dissimilarity: distances, criterion: metric stress) in 2D of (A) horses, (B) multiple animal categories, and (C) rabbits, and also (D) the networks correlation with human arrangements for horse (purple), rabbit (yellow), and multiple animal silhouettes (gray). While the network does show some groupings based on object category (e.g, spiders in B), overall the network correlates poorly to human similarity arrangements. In comparison, see human and ShapeComp in Figure 2 of manuscript.

For the same reason mentioned above, meaning that, perception does not happen in a vacuum, I feel that reducing the input to shape outline only, might be limiting the potentiality of such a model. I wonder whether including additional descriptors that capture information from the object line drawing, which provide important shape features, could better capture more realistic human shape perception abilities. Two different objects might have the same outline when seen from a specific viewpoint but could nevertheless be distinguishable if depicted with line drawing. To be honest, the authors already discuss some of the model's limitations in similar directions (e.g., 3D object information). However, I would appreciate a further discussion of aspects that take into consideration real object perception.

Yes, indeed. There are many other potential cues that could be added to capture more complex aspects of human shape perception. We have added to our discussion of this on page 38:

Second, even highly reduced line drawings often provide additional cues for disambiguating form within the silhouette boundary (Koenderink, 1984; Malik, 1987; Koenderink et al, 1992, 1996; Judd et al 2007; Cole et al 2008, 2009; Kunsberg et al 2018). For example, Pinna (2010) showed how adding context such as inner line drawings could change our shape percepts as arising from one to two distinct objects. In addition, Wilder et al. (2019) showed how symmetry within local contours of line drawings facilitates human scene categorization (See also: Damiano et al., 2019; Rezanejad et al., 2019). Thus, there are many other ways to derive additional information from line drawings within a scene, in addition to the shape's silhouette, which are important for the coding of shape.

It is interesting to see that when combining multiple shape descriptors the model can reach good performance regardless of what subset of dimensions is considered. I agree with the authors that this should not be taken as evidence for equal importance/contribution of the descriptors but shows that the descriptors inevitably overlap to a certain degree and if many descriptors are considered it becomes less relevant what specific subset is selected. I wonder whether this might also be a consequence of the input choice, as mentioned above; using object outlines reduces the available information, possibly resulting in the different descriptors being more correlated among each other. This is not a critique, is just a thought. Am curious to know the authors' thoughts on this point.

Thanks for the suggestion; this is an interesting point to consider in our future work. While many shape descriptors will be correlated and also tap into similar kinds of information, the descriptors are not identical. One can already see by comparing Figure 1E and S1 that transforming the shapes in different ways shifts the relative sensitivity of shape descriptors in different ways. Thus, we agree that with more information, not just object outlines, it is possible that the descriptors would be less correlated or that some shape descriptors would become less correlated and others more correlated.

We could, for example, also investigate how the shape descriptors vary across shapes from different generative processes (e.g., man-made vs. natural). Given that shapes from different processes will vary, looking at changes in correlations between shape features could be a good way of looking at which features predict a particular generative process. We look forward to exploring this in greater depth in follow-up studies.
